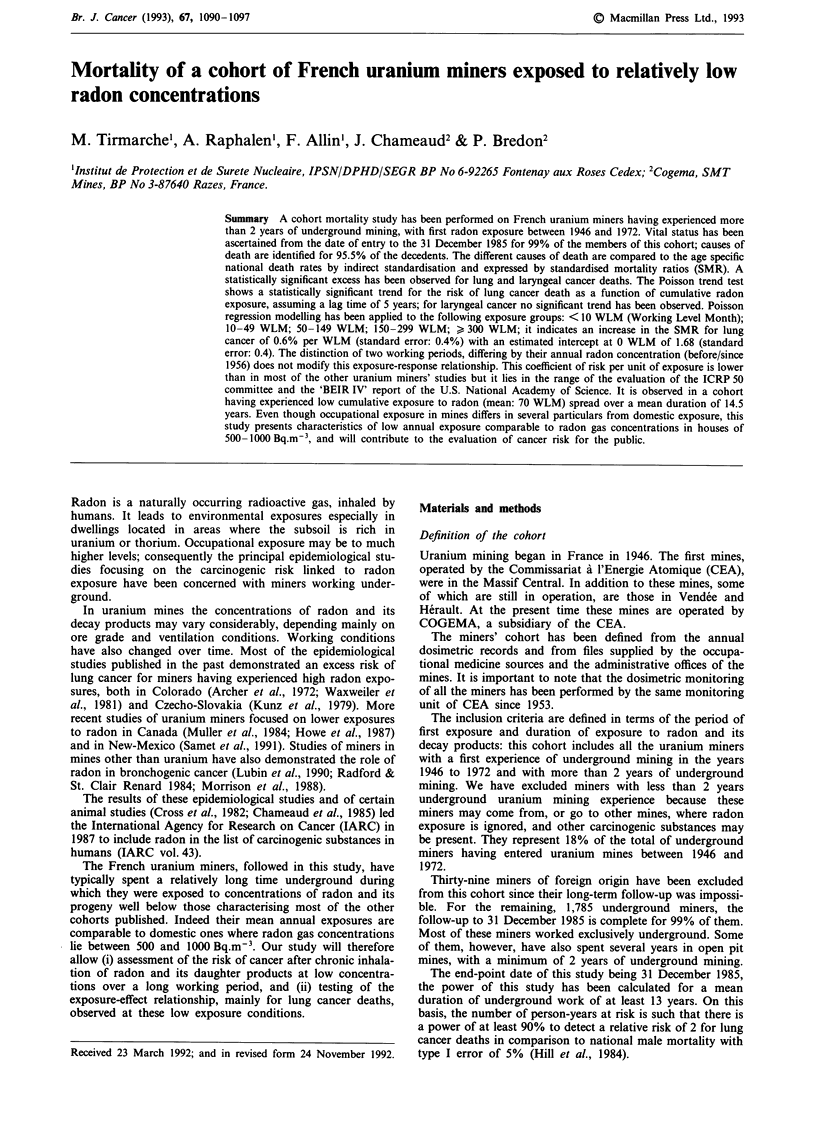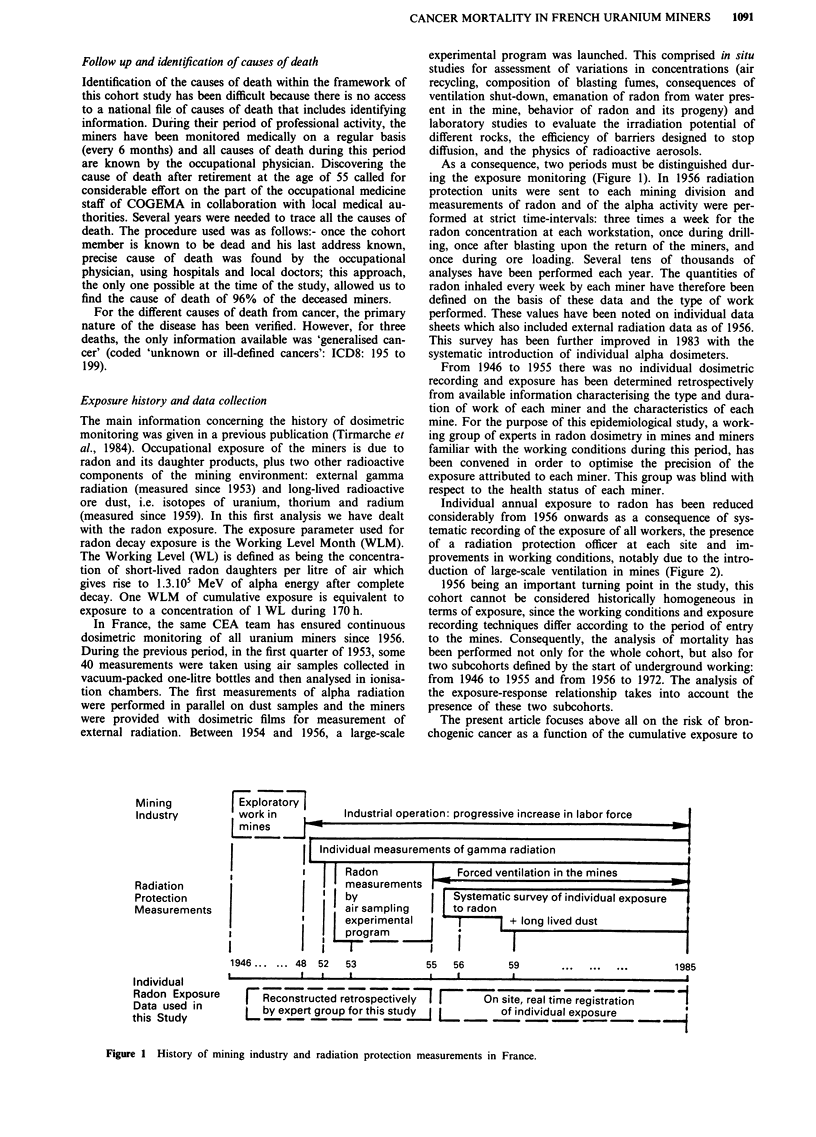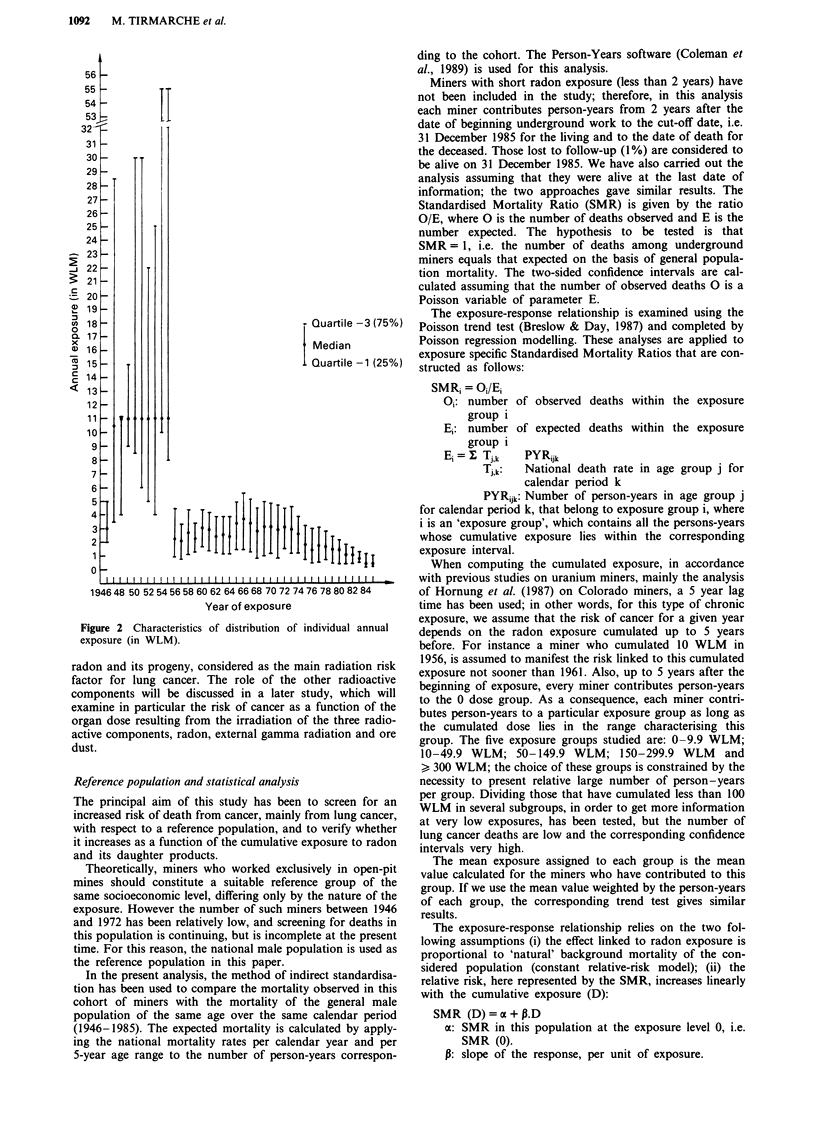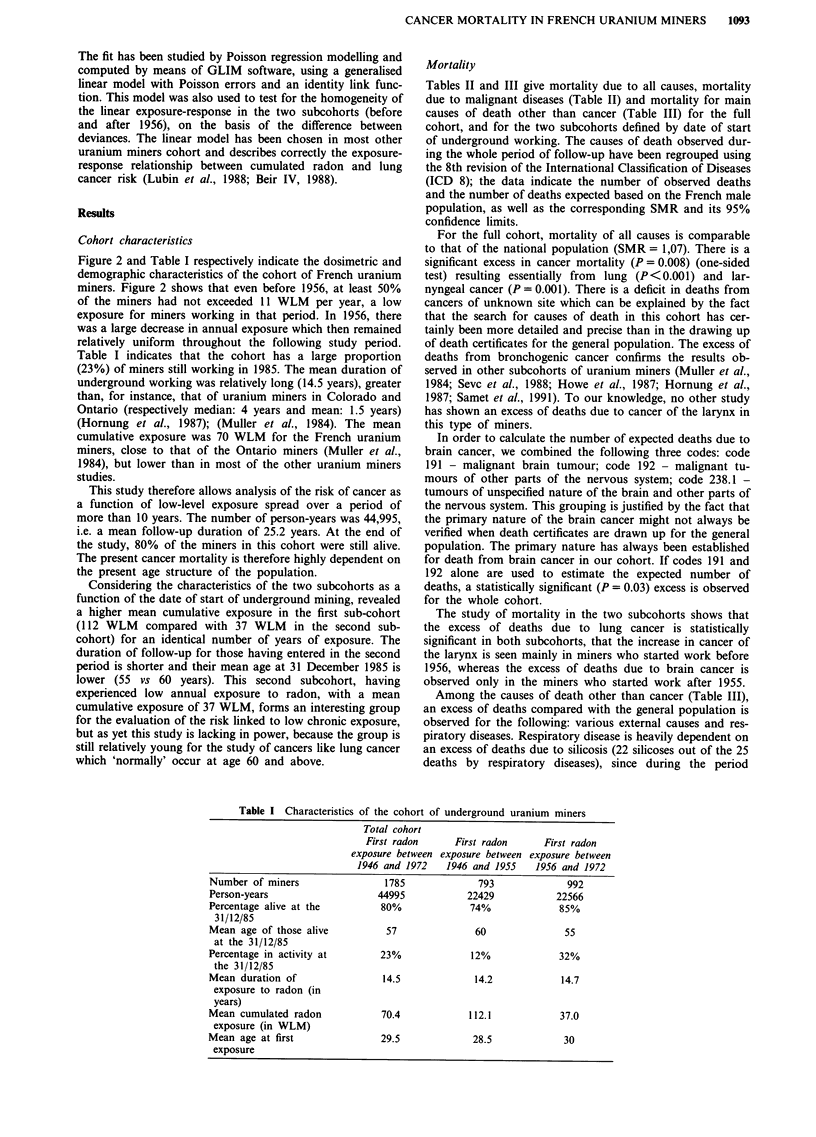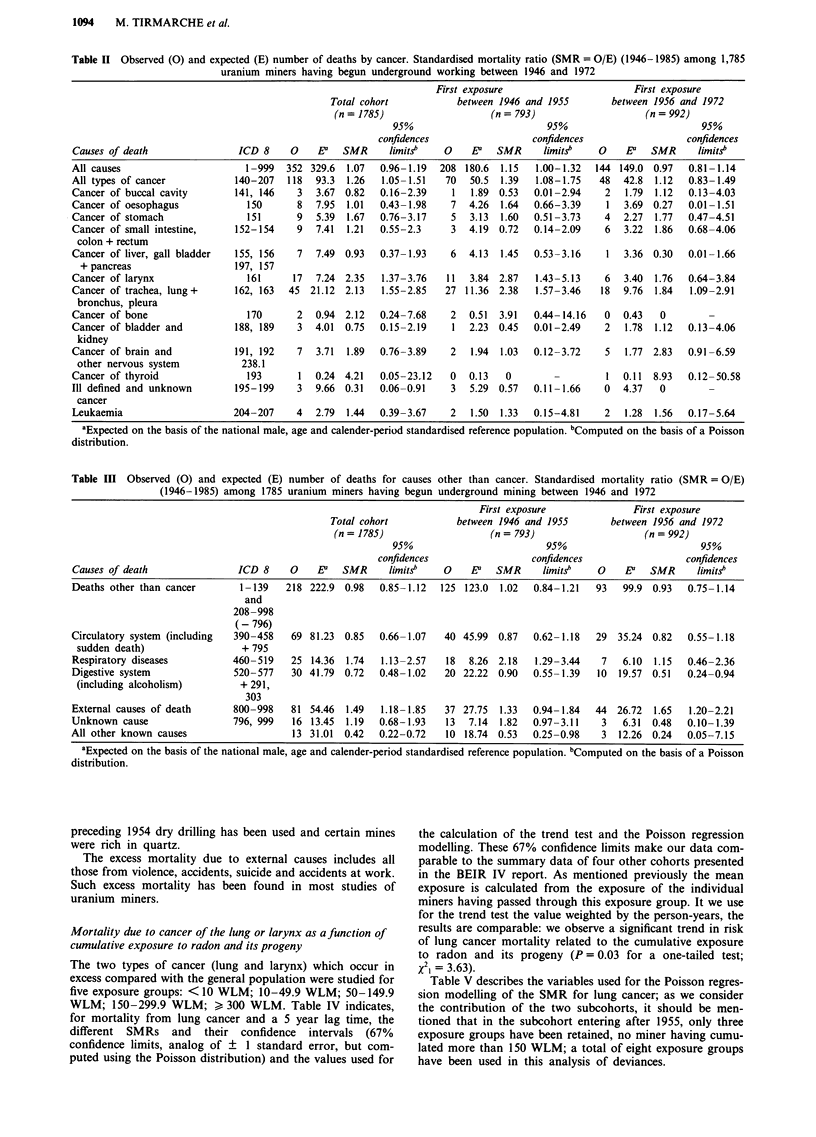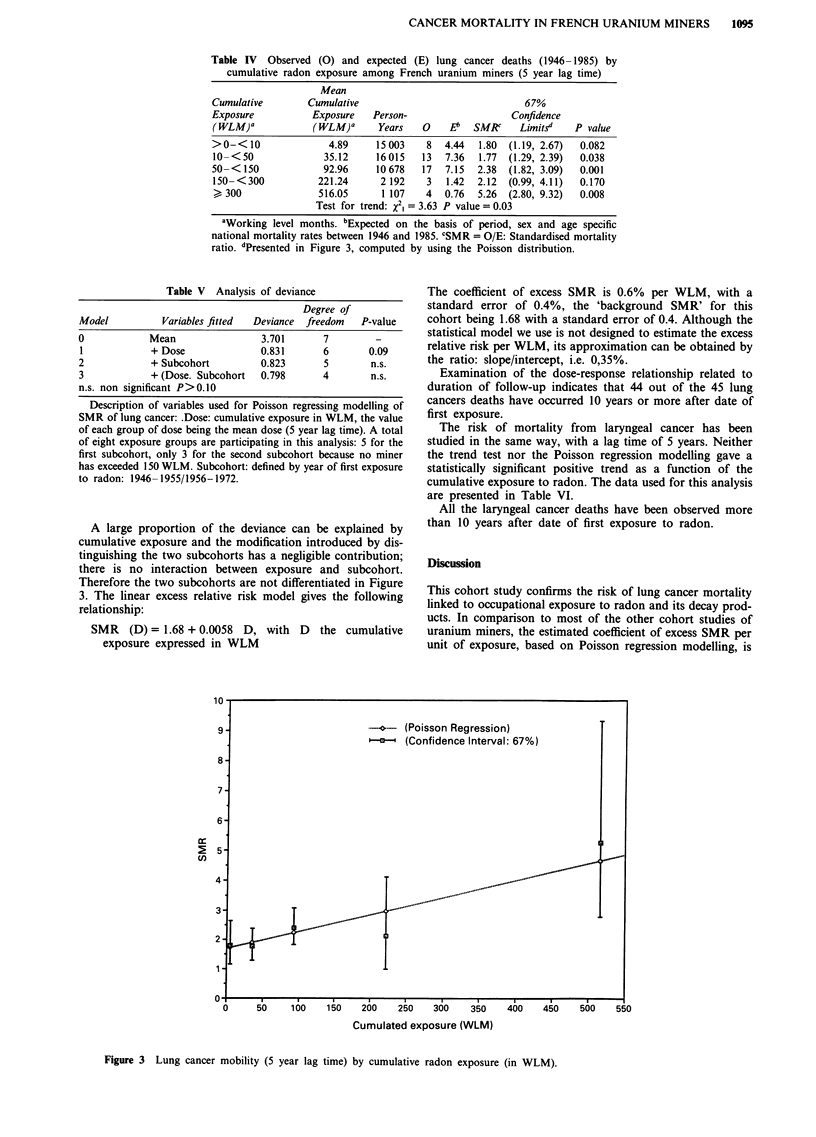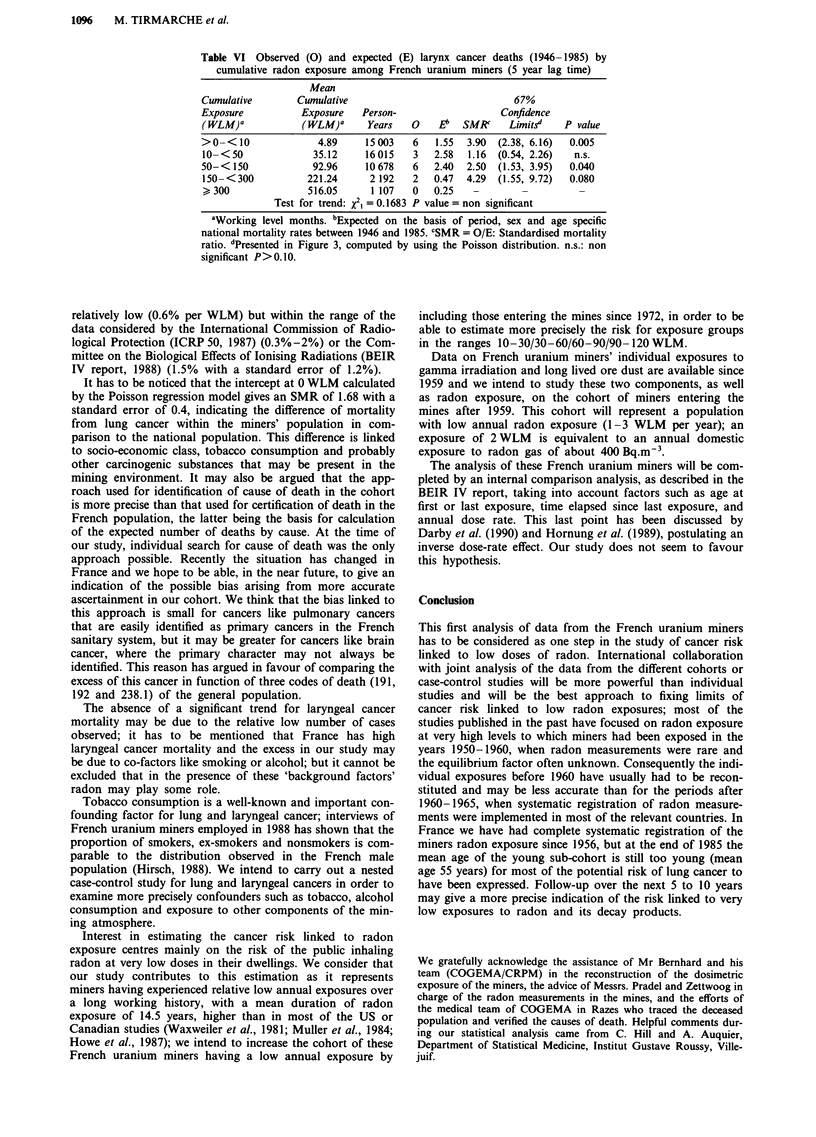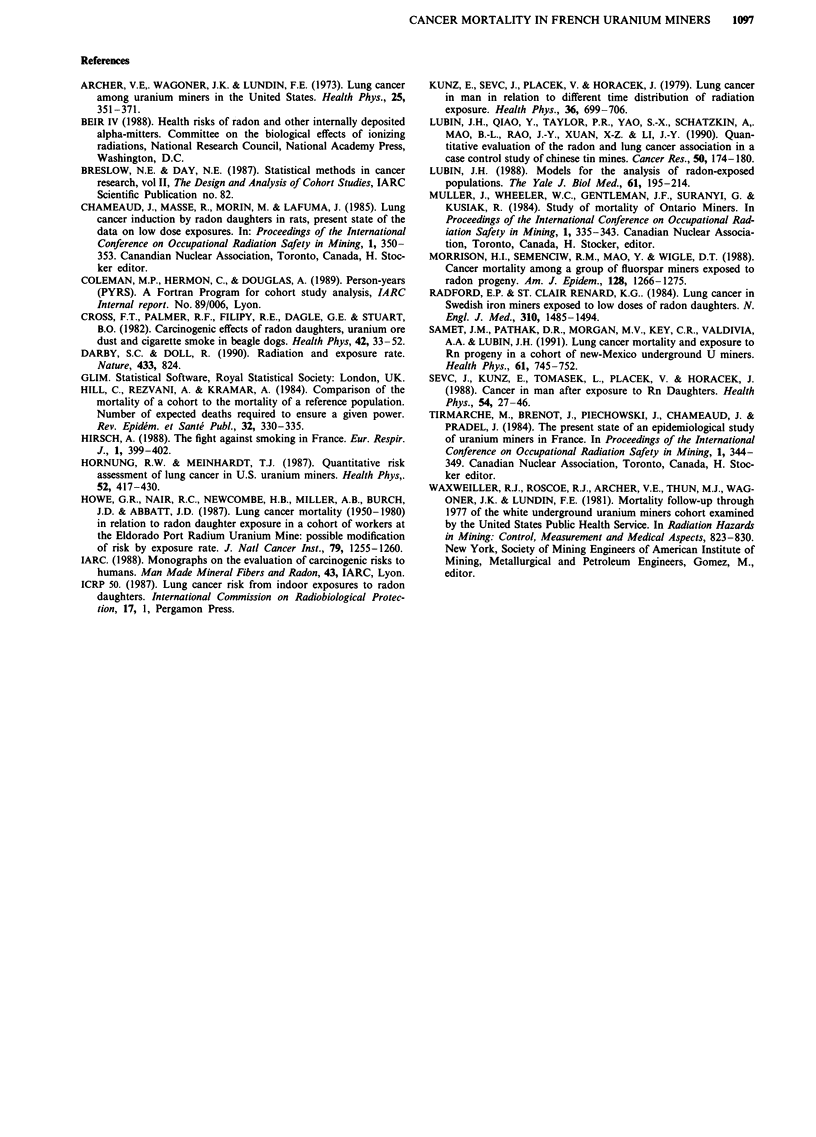# Mortality of a cohort of French uranium miners exposed to relatively low radon concentrations.

**DOI:** 10.1038/bjc.1993.200

**Published:** 1993-05

**Authors:** M. Tirmarche, A. Raphalen, F. Allin, J. Chameaud, P. Bredon

**Affiliations:** Institut de Protection et de Surete Nucleaire, Fontenay aux Roses, France.

## Abstract

A cohort mortality study has been performed on French uranium miners having experienced more than 2 years of underground mining, with first radon exposure between 1946 and 1972. Vital status has been ascertained from the date of entry to the 31 December 1985 for 99% of the members of this cohort; causes of death are identified for 95.5% of the decedents. The different causes of death are compared to the age specific national death rates by indirect standardisation and expressed by standardised mortality ratios (SMR). A statistically significant excess has been observed for lung and laryngeal cancer deaths. The Poisson trend test shows a statistically significant trend for the risk of lung cancer death as a function of cumulative radon exposure, assuming a lag time of 5 years; for laryngeal cancer no significant trend has been observed. Poisson regression modelling has been applied to the following exposure groups: < 10 WLM (Working Level Month); 10-49 WLM; 50-149 WLM; 150-299 WLM; > or = 300 WLM; it indicates an increase in the SMR for lung cancer of 0.6% per WLM (standard error: 0.4%) with an estimated intercept at 0 WLM of 1.68 (standard error: 0.4). The distinction of two working periods, differing by their annual radon concentration (before/since 1956) does not modify this exposure-response relationship. This coefficient of risk per unit of exposure is lower than in most of the other uranium miners' studies but it lies in the range of the evaluation of the ICRP 50 committee and the 'BEIR IV' report of the U.S. National Academy of Science. It is observed in a cohort having experienced low cumulative exposure to radon (mean: 70 WLM) spread over a mean duration of 14.5 years. Even though occupational exposure in mines differs in several particulars from domestic exposure, this study presents characteristics of low annual exposure comparable to radon gas concentrations in houses of 500-1000 Bq.m-3, and will contribute to the evaluation of cancer risk for the public.


Br. J. Cancer (1993), 67, 1090-1097 © Macmillan Press Ltd., 1993

### Mortality of a cohort of French uranium miners exposed to relatively low radon concentrations

M. Tirmarche[1], A. Raphalen[1], F. Allin[1], J. Chameaud[2] & P. Bredon[2]

[1]*Institut de Protection et de Surete Nucleaire, IPSN/DPHD/SEGR BP No 6-92265 Fontenay aux Roses Cedex;* [2]*Cogema, SMT Mines, BP No 3-87640 Razes, France.*

**Summary** A cohort mortality study has been performed on French uranium miners having experienced more than 2 years of underground mining, with first radon exposure between 1946 and 1972. Vital status has been ascertained from the date of entry to the 31 December 1985 for 99% of the members of this cohort; causes of death are identified for 95.5% of the decedents. The different causes of death are compared to the age specific national death rates by indirect standardisation and expressed by standardised mortality ratios (SMR). A statistically significant excess has been observed for lung and laryngeal cancer deaths. The Poisson trend test shows a statistically significant trend for the risk of lung cancer death as a function of cumulative radon exposure, assuming a lag time of 5 years; for laryngeal cancer no significant trend has been observed. Poisson regression modelling has been applied to the following exposure groups: $<10$ WLM (Working Level Month); 10-49 WLM; 50-149 WLM; 150-299 WLM; $\geqslant 300$ WLM; it indicates an increase in the SMR for lung cancer of 0.6% per WLM (standard error: 0.4%) with an estimated intercept at 0 WLM of 1.68 (standard error: 0.4). The distinction of two working periods, differing by their annual radon concentration (before/since 1956) does not modify this exposure-response relationship. This coefficient of risk per unit of exposure is lower than in most of the other uranium miners' studies but it lies in the range of the evaluation of the ICRP 50 committee and the 'BEIR IV' report of the U.S. National Academy of Science. It is observed in a cohort having experienced low cumulative exposure to radon (mean: 70 WLM) spread over a mean duration of 14.5 years. Even though occupational exposure in mines differs in several particulars from domestic exposure, this study presents characteristics of low annual exposure comparable to radon gas concentrations in houses of 500-1000 $Bq.m^{-3}$, and will contribute to the evaluation of cancer risk for the public.

Radon is a naturally occurring radioactive gas, inhaled by humans. It leads to environmental exposures especially in dwellings located in areas where the subsoil is rich in uranium or thorium. Occupational exposure may be to much higher levels; consequently the principal epidemiological studies focusing on the carcinogenic risk linked to radon exposure have been concerned with miners working underground.

In uranium mines the concentrations of radon and its decay products may vary considerably, depending mainly on ore grade and ventilation conditions. Working conditions have also changed over time. Most of the epidemiological studies published in the past demonstrated an excess risk of lung cancer for miners having experienced high radon exposures, both in Colorado (Archer *et al.*, 1972; Waxweiler *et al.*, 1981) and Czecho-Slovakia (Kunz *et al.*, 1979). More recent studies of uranium miners focused on lower exposures to radon in Canada (Muller *et al.*, 1984; Howe *et al.*, 1987) and in New-Mexico (Samet *et al.*, 1991). Studies of miners in mines other than uranium have also demonstrated the role of radon in bronchogenic cancer (Lubin *et al.*, 1990; Radford & St. Clair Renard 1984; Morrison *et al.*, 1988).

The results of these epidemiological studies and of certain animal studies (Cross *et al.*, 1982; Chameaud *et al.*, 1985) led the International Agency for Research on Cancer (IARC) in 1987 to include radon in the list of carcinogenic substances in humans (IARC vol. 43).

The French uranium miners, followed in this study, have typically spent a relatively long time underground during which they were exposed to concentrations of radon and its progeny well below those characterising most of the other cohorts published. Indeed their mean annual exposures are comparable to domestic ones where radon gas concentrations lie between 500 and 1000 $Bq.m^{-3}$. Our study will therefore allow (i) assessment of the risk of cancer after chronic inhalation of radon and its daughter products at low concentrations over a long working period, and (ii) testing of the exposure-effect relationship, mainly for lung cancer deaths, observed at these low exposure conditions.

Received 23 March 1992; and in revised form 24 November 1992.

### Materials and methods

### *Definition of the cohort*

Uranium mining began in France in 1946. The first mines, operated by the Commissariat à l'Energie Atomique (CEA), were in the Massif Central. In addition to these mines, some of which are still in operation, are those in Vendée and Hérault. At the present time these mines are operated by COGEMA, a subsidiary of the CEA.

The miners' cohort has been defined from the annual dosimetric records and from files supplied by the occupational medicine sources and the administrative offices of the mines. It is important to note that the dosimetric monitoring of all the miners has been performed by the same monitoring unit of CEA since 1953.

The inclusion criteria are defined in terms of the period of first exposure and duration of exposure to radon and its decay products: this cohort includes all the uranium miners with a first experience of underground mining in the years 1946 to 1972 and with more than 2 years of underground mining. We have excluded miners with less than 2 years underground uranium mining experience because these miners may come from, or go to other mines, where radon exposure is ignored, and other carcinogenic substances may be present. They represent 18% of the total of underground miners having entered uranium mines between 1946 and 1972.

Thirty-nine miners of foreign origin have been excluded from this cohort since their long-term follow-up was impossible. For the remaining, 1,785 underground miners, the follow-up to 31 December 1985 is complete for 99% of them. Most of these miners worked exclusively underground. Some of them, however, have also spent several years in open pit mines, with a minimum of 2 years of underground mining.

The end-point date of this study being 31 December 1985, the power of this study has been calculated for a mean duration of underground work of at least 13 years. On this basis, the number of person-years at risk is such that there is a power of at least 90% to detect a relative risk of 2 for lung cancer deaths in comparison to national male mortality with type I error of 5% (Hill *et al.*, 1984).

*Follow up and identification of causes of death*

Identification of the causes of death within the framework of this cohort study has been difficult because there is no access to a national file of causes of death that includes identifying information. During their period of professional activity, the miners have been monitored medically on a regular basis (every 6 months) and all causes of death during this period are known by the occupational physician. Discovering the cause of death after retirement at the age of 55 called for considerable effort on the part of the occupational medicine staff of COGEMA in collaboration with local medical authorities. Several years were needed to trace all the causes of death. The procedure used was as follows:- once the cohort member is known to be dead and his last address known, precise cause of death was found by the occupational physician, using hospitals and local doctors; this approach, the only one possible at the time of the study, allowed us to find the cause of death of 96% of the deceased miners.

For the different causes of death from cancer, the primary nature of the disease has been verified. However, for three deaths, the only information available was 'generalised cancer' (coded 'unknown or ill-defined cancers': ICD8: 195 to 199).

*Exposure history and data collection*

The main information concerning the history of dosimetric monitoring was given in a previous publication (Tirmarche *et al.*, 1984). Occupational exposure of the miners is due to radon and its daughter products, plus two other radioactive components of the mining environment: external gamma radiation (measured since 1953) and long-lived radioactive ore dust, i.e. isotopes of uranium, thorium and radium (measured since 1959). In this first analysis we have dealt with the radon exposure. The exposure parameter used for radon decay exposure is the Working Level Month (WLM). The Working Level (WL) is defined as being the concentration of short-lived radon daughters per litre of air which gives rise to $1.3.10^5$ MeV of alpha energy after complete decay. One WLM of cumulative exposure is equivalent to exposure to a concentration of 1 WL during 170 h.

In France, the same CEA team has ensured continuous dosimetric monitoring of all uranium miners since 1956. During the previous period, in the first quarter of 1953, some 40 measurements were taken using air samples collected in vacuum-packed one-litre bottles and then analysed in ionisation chambers. The first measurements of alpha radiation were performed in parallel on dust samples and the miners were provided with dosimetric films for measurement of external radiation. Between 1954 and 1956, a large-scale experimental program was launched. This comprised *in situ* studies for assessment of variations in concentrations (air recycling, composition of blasting fumes, consequences of ventilation shut-down, emanation of radon from water present in the mine, behavior of radon and its progeny) and laboratory studies to evaluate the irradiation potential of different rocks, the efficiency of barriers designed to stop diffusion, and the physics of radioactive aerosols.

As a consequence, two periods must be distinguished during the exposure monitoring (Figure 1). In 1956 radiation protection units were sent to each mining division and measurements of radon and of the alpha activity were performed at strict time-intervals: three times a week for the radon concentration at each workstation, once during drilling, once after blasting upon the return of the miners, and once during ore loading. Several tens of thousands of analyses have been performed each year. The quantities of radon inhaled every week by each miner have therefore been defined on the basis of these data and the type of work performed. These values have been noted on individual data sheets which also included external radiation data as of 1956. This survey has been further improved in 1983 with the systematic introduction of individual alpha dosimeters.

From 1946 to 1955 there was no individual dosimetric recording and exposure has been determined retrospectively from available information characterising the type and duration of work of each miner and the characteristics of each mine. For the purpose of this epidemiological study, a working group of experts in radon dosimetry in mines and miners familiar with the working conditions during this period, has been convened in order to optimise the precision of the exposure attributed to each miner. This group was blind with respect to the health status of each miner.

Individual annual exposure to radon has been reduced considerably from 1956 onwards as a consequence of systematic recording of the exposure of all workers, the presence of a radiation protection officer at each site and improvements in working conditions, notably due to the introduction of large-scale ventilation in mines (Figure 2).

1956 being an important turning point in the study, this cohort cannot be considered historically homogeneous in terms of exposure, since the working conditions and exposure recording techniques differ according to the period of entry to the mines. Consequently, the analysis of mortality has been performed not only for the whole cohort, but also for two subcohorts defined by the start of underground working: from 1946 to 1955 and from 1956 to 1972. The analysis of the exposure-response relationship takes into account the presence of these two subcohorts.

The present article focuses above all on the risk of bronchogenic cancer as a function of the cumulative exposure to

**Figure 1** History of mining industry and radiation protection measurements in France.

**Figure 2** Characteristics of distribution of individual annual exposure (in WLM).

radon and its progeny, considered as the main radiation risk factor for lung cancer. The role of the other radioactive components will be discussed in a later study, which will examine in particular the risk of cancer as a function of the organ dose resulting from the irradiation of the three radioactive components, radon, external gamma radiation and ore dust.

*Reference population and statistical analysis*

The principal aim of this study has been to screen for an increased risk of death from cancer, mainly from lung cancer, with respect to a reference population, and to verify whether it increases as a function of the cumulative exposure to radon and its daughter products.

Theoretically, miners who worked exclusively in open-pit mines should constitute a suitable reference group of the same socioeconomic level, differing only by the nature of the exposure. However the number of such miners between 1946 and 1972 has been relatively low, and screening for deaths in this population is continuing, but is incomplete at the present time. For this reason, the national male population is used as the reference population in this paper.

In the present analysis, the method of indirect standardisation has been used to compare the mortality observed in this cohort of miners with the mortality of the general male population of the same age over the same calendar period (1946–1985). The expected mortality is calculated by applying the national mortality rates per calendar year and per 5-year age range to the number of person-years corresponding to the cohort. The Person-Years software (Coleman *et al.*, 1989) is used for this analysis.

Miners with short radon exposure (less than 2 years) have not been included in the study; therefore, in this analysis each miner contributes person-years from 2 years after the date of beginning underground work to the cut-off date, i.e. 31 December 1985 for the living and to the date of death for the deceased. Those lost to follow-up (1%) are considered to be alive on 31 December 1985. We have also carried out the analysis assuming that they were alive at the last date of information; the two approaches gave similar results. The Standardised Mortality Ratio (SMR) is given by the ratio O/E, where O is the number of deaths observed and E is the number expected. The hypothesis to be tested is that SMR = 1, i.e. the number of deaths among underground miners equals that expected on the basis of general population mortality. The two-sided confidence intervals are calculated assuming that the number of observed deaths O is a Poisson variable of parameter E.

The exposure-response relationship is examined using the Poisson trend test (Breslow & Day, 1987) and completed by Poisson regression modelling. These analyses are applied to exposure specific Standardised Mortality Ratios that are constructed as follows:

$SMR_i = O_i/E_i$

$O_i$: number of observed deaths within the exposure group i

$E_i$: number of expected deaths within the exposure group i

$E_i = \Sigma\ T_{j,k}\quad PYR_{ijk}$

$T_{j,k}$: National death rate in age group j for calendar period k

$PYR_{ijk}$: Number of person-years in age group j for calendar period k, that belong to exposure group i, where i is an 'exposure group', which contains all the persons-years whose cumulative exposure lies within the corresponding exposure interval.

When computing the cumulated exposure, in accordance with previous studies on uranium miners, mainly the analysis of Hornung *et al.* (1987) on Colorado miners, a 5 year lag time has been used; in other words, for this type of chronic exposure, we assume that the risk of cancer for a given year depends on the radon exposure cumulated up to 5 years before. For instance a miner who cumulated 10 WLM in 1956, is assumed to manifest the risk linked to this cumulated exposure not sooner than 1961. Also, up to 5 years after the beginning of exposure, every miner contributes person-years to the 0 dose group. As a consequence, each miner contributes person-years to a particular exposure group as long as the cumulated dose lies in the range characterising this group. The five exposure groups studied are: 0–9.9 WLM; 10–49.9 WLM; 50–149.9 WLM; 150–299.9 WLM and ⩾ 300 WLM; the choice of these groups is constrained by the necessity to present relative large number of person–years per group. Dividing those that have cumulated less than 100 WLM in several subgroups, in order to get more information at very low exposures, has been tested, but the number of lung cancer deaths are low and the corresponding confidence intervals very high.

The mean exposure assigned to each group is the mean value calculated for the miners who have contributed to this group. If we use the mean value weighted by the person-years of each group, the corresponding trend test gives similar results.

The exposure-response relationship relies on the two following assumptions (i) the effect linked to radon exposure is proportional to 'natural' background mortality of the considered population (constant relative-risk model); (ii) the relative risk, here represented by the SMR, increases linearly with the cumulative exposure (D):

$SMR\ (D) = \alpha + \beta.D$

$\alpha$: SMR in this population at the exposure level 0, i.e. SMR (0).

$\beta$: slope of the response, per unit of exposure.

The fit has been studied by Poisson regression modelling and computed by means of GLIM software, using a generalised linear model with Poisson errors and an identity link function. This model was also used to test for the homogeneity of the linear exposure-response in the two subcohorts (before and after 1956), on the basis of the difference between deviances. The linear model has been chosen in most other uranium miners cohort and describes correctly the exposure-response relationship between cumulated radon and lung cancer risk (Lubin *et al.*, 1988; Beir IV, 1988).

### Results

### *Cohort characteristics*

Figure 2 and Table I respectively indicate the dosimetric and demographic characteristics of the cohort of French uranium miners. Figure 2 shows that even before 1956, at least 50% of the miners had not exceeded 11 WLM per year, a low exposure for miners working in that period. In 1956, there was a large decrease in annual exposure which then remained relatively uniform throughout the following study period. Table I indicates that the cohort has a large proportion (23%) of miners still working in 1985. The mean duration of underground working was relatively long (14.5 years), greater than, for instance, that of uranium miners in Colorado and Ontario (respectively median: 4 years and mean: 1.5 years) (Hornung *et al.*, 1987); (Muller *et al.*, 1984). The mean cumulative exposure was 70 WLM for the French uranium miners, close to that of the Ontario miners (Muller *et al.*, 1984), but lower than in most of the other uranium miners studies.

This study therefore allows analysis of the risk of cancer as a function of low-level exposure spread over a period of more than 10 years. The number of person-years was 44,995, i.e. a mean follow-up duration of 25.2 years. At the end of the study, 80% of the miners in this cohort were still alive. The present cancer mortality is therefore highly dependent on the present age structure of the population.

Considering the characteristics of the two subcohorts as a function of the date of start of underground mining, revealed a higher mean cumulative exposure in the first sub-cohort (112 WLM compared with 37 WLM in the second sub-cohort) for an identical number of years of exposure. The duration of follow-up for those having entered in the second period is shorter and their mean age at 31 December 1985 is lower (55 *vs* 60 years). This second subcohort, having experienced low annual exposure to radon, with a mean cumulative exposure of 37 WLM, forms an interesting group for the evaluation of the risk linked to low chronic exposure, but as yet this study is lacking in power, because the group is still relatively young for the study of cancers like lung cancer which 'normally' occur at age 60 and above.

### *Mortality*

Tables II and III give mortality due to all causes, mortality due to malignant diseases (Table II) and mortality for main causes of death other than cancer (Table III) for the full cohort, and for the two subcohorts defined by date of start of underground working. The causes of death observed during the whole period of follow-up have been regrouped using the 8th revision of the International Classification of Diseases (ICD 8); the data indicate the number of observed deaths and the number of deaths expected based on the French male population, as well as the corresponding SMR and its 95% confidence limits.

For the full cohort, mortality of all causes is comparable to that of the national population (SMR = 1,07). There is a significant excess in cancer mortality ($P = 0.008$) (one-sided test) resulting essentially from lung ($P < 0.001$) and laryngeal cancer ($P = 0.001$). There is a deficit in deaths from cancers of unknown site which can be explained by the fact that the search for causes of death in this cohort has certainly been more detailed and precise than in the drawing up of death certificates for the general population. The excess of deaths from bronchogenic cancer confirms the results observed in other subcohorts of uranium miners (Muller *et al.*, 1984; Sevc *et al.*, 1988; Howe *et al.*, 1987; Hornung *et al.*, 1987; Samet *et al.*, 1991). To our knowledge, no other study has shown an excess of deaths due to cancer of the larynx in this type of miners.

In order to calculate the number of expected deaths due to brain cancer, we combined the following three codes: code 191 – malignant brain tumour; code 192 – malignant tumours of other parts of the nervous system; code 238.1 – tumours of unspecified nature of the brain and other parts of the nervous system. This grouping is justified by the fact that the primary nature of the brain cancer might not always be verified when death certificates are drawn up for the general population. The primary nature has always been established for death from brain cancer in our cohort. If codes 191 and 192 alone are used to estimate the expected number of deaths, a statistically significant ($P = 0.03$) excess is observed for the whole cohort.

The study of mortality in the two subcohorts shows that the excess of deaths due to lung cancer is statistically significant in both subcohorts, that the increase in cancer of the larynx is seen mainly in miners who started work before 1956, whereas the excess of deaths due to brain cancer is observed only in the miners who started work after 1955.

Among the causes of death other than cancer (Table III), an excess of deaths compared with the general population is observed for the following: various external causes and respiratory diseases. Respiratory disease is heavily dependent on an excess of deaths due to silicosis (22 silicoses out of the 25 deaths by respiratory diseases), since during the period

**Table I** Characteristics of the cohort of underground uranium miners

| | *Total cohort First radon exposure between 1946 and 1972* | *First radon exposure between 1946 and 1955* | *First radon exposure between 1956 and 1972* |
|---|---|---|---|
| Number of miners | 1785 | 793 | 992 |
| Person-years | 44995 | 22429 | 22566 |
| Percentage alive at the 31/12/85 | 80% | 74% | 85% |
| Mean age of those alive at the 31/12/85 | 57 | 60 | 55 |
| Percentage in activity at the 31/12/85 | 23% | 12% | 32% |
| Mean duration of exposure to radon (in years) | 14.5 | 14.2 | 14.7 |
| Mean cumulated radon exposure (in WLM) | 70.4 | 112.1 | 37.0 |
| Mean age at first exposure | 29.5 | 28.5 | 30 |

**Table II** Observed (O) and expected (E) number of deaths by cancer. Standardised mortality ratio (SMR = O/E) (1946–1985) among 1,785 uranium miners having begun underground working between 1946 and 1972

| | | *Total cohort (n = 1785)* | | | | *First exposure between 1946 and 1955 (n = 793)* | | | | *First exposure between 1956 and 1972 (n = 992)* | | | |
|---|---|---|---|---|---|---|---|---|---|---|---|---|---|
| *Causes of death* | *ICD 8* | *O* | *E*[a] | *SMR* | *95% confidences limits*[b] | *O* | *E*[a] | *SMR* | *95% confidences limits*[b] | *O* | *E*[a] | *SMR* | *95% confidences limits*[b] |
| All causes | 1–999 | 352 | 329.6 | 1.07 | 0.96–1.19 | 208 | 180.6 | 1.15 | 1.00–1.32 | 144 | 149.0 | 0.97 | 0.81–1.14 |
| All types of cancer | 140–207 | 118 | 93.3 | 1.26 | 1.05–1.51 | 70 | 50.5 | 1.39 | 1.08–1.75 | 48 | 42.8 | 1.12 | 0.83–1.49 |
| Cancer of buccal cavity | 141, 146 | 3 | 3.67 | 0.82 | 0.16–2.39 | 1 | 1.89 | 0.53 | 0.01–2.94 | 2 | 1.79 | 1.12 | 0.13–4.03 |
| Cancer of oesophagus | 150 | 8 | 7.95 | 1.01 | 0.43–1.98 | 7 | 4.26 | 1.64 | 0.66–3.39 | 1 | 3.69 | 0.27 | 0.01–1.51 |
| Cancer of stomach | 151 | 9 | 5.39 | 1.67 | 0.76–3.17 | 5 | 3.13 | 1.60 | 0.51–3.73 | 4 | 2.27 | 1.77 | 0.47–4.51 |
| Cancer of small intestine, colon + rectum | 152–154 | 9 | 7.41 | 1.21 | 0.55–2.3 | 3 | 4.19 | 0.72 | 0.14–2.09 | 6 | 3.22 | 1.86 | 0.68–4.06 |
| Cancer of liver, gall bladder + pancreas | 155, 156 197, 157 | 7 | 7.49 | 0.93 | 0.37–1.93 | 6 | 4.13 | 1.45 | 0.53–3.16 | 1 | 3.36 | 0.30 | 0.01–1.66 |
| Cancer of larynx | 161 | 17 | 7.24 | 2.35 | 1.37–3.76 | 11 | 3.84 | 2.87 | 1.43–5.13 | 6 | 3.40 | 1.76 | 0.64–3.84 |
| Cancer of trachea, lung + bronchus, pleura | 162, 163 | 45 | 21.12 | 2.13 | 1.55–2.85 | 27 | 11.36 | 2.38 | 1.57–3.46 | 18 | 9.76 | 1.84 | 1.09–2.91 |
| Cancer of bone | 170 | 2 | 0.94 | 2.12 | 0.24–7.68 | 2 | 0.51 | 3.91 | 0.44–14.16 | 0 | 0.43 | 0 | – |
| Cancer of bladder and kidney | 188, 189 | 3 | 4.01 | 0.75 | 0.15–2.19 | 1 | 2.23 | 0.45 | 0.01–2.49 | 2 | 1.78 | 1.12 | 0.13–4.06 |
| Cancer of brain and other nervous system | 191, 192 238.1 | 7 | 3.71 | 1.89 | 0.76–3.89 | 2 | 1.94 | 1.03 | 0.12–3.72 | 5 | 1.77 | 2.83 | 0.91–6.59 |
| Cancer of thyroid | 193 | 1 | 0.24 | 4.21 | 0.05–23.12 | 0 | 0.13 | 0 | – | 1 | 0.11 | 8.93 | 0.12–50.58 |
| Ill defined and unknown cancer | 195–199 | 3 | 9.66 | 0.31 | 0.06–0.91 | 3 | 5.29 | 0.57 | 0.11–1.66 | 0 | 4.37 | 0 | – |
| Leukaemia | 204–207 | 4 | 2.79 | 1.44 | 0.39–3.67 | 2 | 1.50 | 1.33 | 0.15–4.81 | 2 | 1.28 | 1.56 | 0.17–5.64 |

[a]Expected on the basis of the national male, age and calender-period standardised reference population. [b]Computed on the basis of a Poisson distribution.

**Table III** Observed (O) and expected (E) number of deaths for causes other than cancer. Standardised mortality ratio (SMR = O/E) (1946–1985) among 1785 uranium miners having begun underground mining between 1946 and 1972

| | | *Total cohort (n = 1785)* | | | | *First exposure between 1946 and 1955 (n = 793)* | | | | *First exposure between 1956 and 1972 (n = 992)* | | | |
|---|---|---|---|---|---|---|---|---|---|---|---|---|---|
| *Causes of death* | *ICD 8* | *O* | *E*[a] | *SMR* | *95% confidences limits*[b] | *O* | *E*[a] | *SMR* | *95% confidences limits*[b] | *O* | *E*[a] | *SMR* | *95% confidences limits*[b] |
| Deaths other than cancer | 1–139 and 208–998 (– 796) | 218 | 222.9 | 0.98 | 0.85–1.12 | 125 | 123.0 | 1.02 | 0.84–1.21 | 93 | 99.9 | 0.93 | 0.75–1.14 |
| Circulatory system (including sudden death) | 390–458 + 795 | 69 | 81.23 | 0.85 | 0.66–1.07 | 40 | 45.99 | 0.87 | 0.62–1.18 | 29 | 35.24 | 0.82 | 0.55–1.18 |
| Respiratory diseases | 460–519 | 25 | 14.36 | 1.74 | 1.13–2.57 | 18 | 8.26 | 2.18 | 1.29–3.44 | 7 | 6.10 | 1.15 | 0.46–2.36 |
| Digestive system (including alcoholism) | 520–577 + 291, 303 | 30 | 41.79 | 0.72 | 0.48–1.02 | 20 | 22.22 | 0.90 | 0.55–1.39 | 10 | 19.57 | 0.51 | 0.24–0.94 |
| External causes of death | 800–998 | 81 | 54.46 | 1.49 | 1.18–1.85 | 37 | 27.75 | 1.33 | 0.94–1.84 | 44 | 26.72 | 1.65 | 1.20–2.21 |
| Unknown cause | 796, 999 | 16 | 13.45 | 1.19 | 0.68–1.93 | 13 | 7.14 | 1.82 | 0.97–3.11 | 3 | 6.31 | 0.48 | 0.10–1.39 |
| All other known causes | | 13 | 31.01 | 0.42 | 0.22–0.72 | 10 | 18.74 | 0.53 | 0.25–0.98 | 3 | 12.26 | 0.24 | 0.05–7.15 |

[a]Expected on the basis of the national male, age and calender-period standardised reference population. [b]Computed on the basis of a Poisson distribution.

preceding 1954 dry drilling has been used and certain mines were rich in quartz.

The excess mortality due to external causes includes all those from violence, accidents, suicide and accidents at work. Such excess mortality has been found in most studies of uranium miners.

*Mortality due to cancer of the lung or larynx as a function of cumulative exposure to radon and its progeny*

The two types of cancer (lung and larynx) which occur in excess compared with the general population were studied for five exposure groups: $<10$ WLM; 10–49.9 WLM; 50–149.9 WLM; 150–299.9 WLM; $\geqslant 300$ WLM. Table IV indicates, for mortality from lung cancer and a 5 year lag time, the different SMRs and their confidence intervals (67% confidence limits, analog of $\pm$ 1 standard error, but computed using the Poisson distribution) and the values used for the calculation of the trend test and the Poisson regression modelling. These 67% confidence limits make our data comparable to the summary data of four other cohorts presented in the BEIR IV report. As mentioned previously the mean exposure is calculated from the exposure of the individual miners having passed through this exposure group. It we use for the trend test the value weighted by the person-years, the results are comparable: we observe a significant trend in risk of lung cancer mortality related to the cumulative exposure to radon and its progeny ($P = 0.03$ for a one-tailed test; $\chi^2_1 = 3.63$).

Table V describes the variables used for the Poisson regression modelling of the SMR for lung cancer; as we consider the contribution of the two subcohorts, it should be mentioned that in the subcohort entering after 1955, only three exposure groups have been retained, no miner having cumulated more than 150 WLM; a total of eight exposure groups have been used in this analysis of deviances.

**Table IV** Observed (O) and expected (E) lung cancer deaths (1946–1985) by cumulative radon exposure among French uranium miners (5 year lag time)

| *Cumulative Exposure (WLM)*[a] | *Mean Cumulative Exposure (WLM)*[a] | *Person-Years* | *O* | *E*[b] | *SMR*[c] | *67% Confidence Limits*[d] | *P value* |
|---|---|---|---|---|---|---|---|
| >0–<10 | 4.89 | 15 003 | 8 | 4.44 | 1.80 | (1.19, 2.67) | 0.082 |
| 10–<50 | 35.12 | 16 015 | 13 | 7.36 | 1.77 | (1.29, 2.39) | 0.038 |
| 50–<150 | 92.96 | 10 678 | 17 | 7.15 | 2.38 | (1.82, 3.09) | 0.001 |
| 150–<300 | 221.24 | 2 192 | 3 | 1.42 | 2.12 | (0.99, 4.11) | 0.170 |
| ⩾300 | 516.05 | 1 107 | 4 | 0.76 | 5.26 | (2.80, 9.32) | 0.008 |
| | Test for trend: $\chi^2_1 = 3.63$ $P$ value = 0.03 | | | | | | |

[a]Working level months. [b]Expected on the basis of period, sex and age specific national mortality rates between 1946 and 1985. [c]SMR = O/E: Standardised mortality ratio. [d]Presented in Figure 3, computed by using the Poisson distribution.

**Table V** Analysis of deviance

| *Model* | *Variables fitted* | *Deviance* | *Degree of freedom* | *P*-value |
|---|---|---|---|---|
| 0 | Mean | 3.701 | 7 | – |
| 1 | + Dose | 0.831 | 6 | 0.09 |
| 2 | + Subcohort | 0.823 | 5 | n.s. |
| 3 | + (Dose. Subcohort | 0.798 | 4 | n.s. |

n.s. non significant $P > 0.10$

Description of variables used for Poisson regressing modelling of SMR of lung cancer: .Dose: cumulative exposure in WLM, the value of each group of dose being the mean dose (5 year lag time). A total of eight exposure groups are participating in this analysis: 5 for the first subcohort, only 3 for the second subcohort because no miner has exceeded 150 WLM. Subcohort: defined by year of first exposure to radon: 1946–1955/1956–1972.

A large proportion of the deviance can be explained by cumulative exposure and the modification introduced by distinguishing the two subcohorts has a negligible contribution; there is no interaction between exposure and subcohort. Therefore the two subcohorts are not differentiated in Figure 3. The linear excess relative risk model gives the following relationship:

SMR (D) = 1.68 + 0.0058 D, with D the cumulative exposure expressed in WLM

The coefficient of excess SMR is 0.6% per WLM, with a standard error of 0.4%, the 'background SMR' for this cohort being 1.68 with a standard error of 0.4. Although the statistical model we use is not designed to estimate the excess relative risk per WLM, its approximation can be obtained by the ratio: slope/intercept, i.e. 0,35%.

Examination of the dose-response relationship related to duration of follow-up indicates that 44 out of the 45 lung cancers deaths have occurred 10 years or more after date of first exposure.

The risk of mortality from laryngeal cancer has been studied in the same way, with a lag time of 5 years. Neither the trend test nor the Poisson regression modelling gave a statistically significant positive trend as a function of the cumulative exposure to radon. The data used for this analysis are presented in Table VI.

All the laryngeal cancer deaths have been observed more than 10 years after date of first exposure to radon.

### Discussion

This cohort study confirms the risk of lung cancer mortality linked to occupational exposure to radon and its decay products. In comparison to most of the other cohort studies of uranium miners, the estimated coefficient of excess SMR per unit of exposure, based on Poisson regression modelling, is

**Figure 3** Lung cancer mobility (5 year lag time) by cumulative radon exposure (in WLM).

**Table VI** Observed (O) and expected (E) larynx cancer deaths (1946–1985) by cumulative radon exposure among French uranium miners (5 year lag time)

| *Cumulative Exposure (WLM)*[a] | *Mean Cumulative Exposure (WLM)*[a] | *Person-Years* | *O* | *E*[b] | *SMR*[c] | *67% Confidence Limits*[d] | *P value* |
|---|---|---|---|---|---|---|---|
| >0–<10 | 4.89 | 15 003 | 6 | 1.55 | 3.90 | (2.38, 6.16) | 0.005 |
| 10–<50 | 35.12 | 16 015 | 3 | 2.58 | 1.16 | (0.54, 2.26) | n.s. |
| 50–<150 | 92.96 | 10 678 | 6 | 2.40 | 2.50 | (1.53, 3.95) | 0.040 |
| 150–<300 | 221.24 | 2 192 | 2 | 0.47 | 4.29 | (1.55, 9.72) | 0.080 |
| ⩾300 | 516.05 | 1 107 | 0 | 0.25 | – | – | – |
| Test for trend: $\chi^2_1 = 0.1683$ $P$ value = non significant | | | | | | | |

[a]Working level months. [b]Expected on the basis of period, sex and age specific national mortality rates between 1946 and 1985. [c]SMR = O/E: Standardised mortality ratio. [d]Presented in Figure 3, computed by using the Poisson distribution. n.s.: non significant $P > 0.10$.

relatively low (0.6% per WLM) but within the range of the data considered by the International Commission of Radiological Protection (ICRP 50, 1987) (0.3%–2%) or the Committee on the Biological Effects of Ionising Radiations (BEIR IV report, 1988) (1.5% with a standard error of 1.2%).

It has to be noticed that the intercept at 0 WLM calculated by the Poisson regression model gives an SMR of 1.68 with a standard error of 0.4, indicating the difference of mortality from lung cancer within the miners' population in comparison to the national population. This difference is linked to socio-economic class, tobacco consumption and probably other carcinogenic substances that may be present in the mining environment. It may also be argued that the approach used for identification of cause of death in the cohort is more precise than that used for certification of death in the French population, the latter being the basis for calculation of the expected number of deaths by cause. At the time of our study, individual search for cause of death was the only approach possible. Recently the situation has changed in France and we hope to be able, in the near future, to give an indication of the possible bias arising from more accurate ascertainment in our cohort. We think that the bias linked to this approach is small for cancers like pulmonary cancers that are easily identified as primary cancers in the French sanitary system, but it may be greater for cancers like brain cancer, where the primary character may not always be identified. This reason has argued in favour of comparing the excess of this cancer in function of three codes of death (191, 192 and 238.1) of the general population.

The absence of a significant trend for laryngeal cancer mortality may be due to the relative low number of cases observed; it has to be mentioned that France has high laryngeal cancer mortality and the excess in our study may be due to co-factors like smoking or alcohol; but it cannot be excluded that in the presence of these 'background factors' radon may play some role.

Tobacco consumption is a well-known and important confounding factor for lung and laryngeal cancer; interviews of French uranium miners employed in 1988 has shown that the proportion of smokers, ex-smokers and nonsmokers is comparable to the distribution observed in the French male population (Hirsch, 1988). We intend to carry out a nested case-control study for lung and laryngeal cancers in order to examine more precisely confounders such as tobacco, alcohol consumption and exposure to other components of the mining atmosphere.

Interest in estimating the cancer risk linked to radon exposure centres mainly on the risk of the public inhaling radon at very low doses in their dwellings. We consider that our study contributes to this estimation as it represents miners having experienced relative low annual exposures over a long working history, with a mean duration of radon exposure of 14.5 years, higher than in most of the US or Canadian studies (Waxweiler *et al.*, 1981; Muller *et al.*, 1984; Howe *et al.*, 1987); we intend to increase the cohort of these French uranium miners having a low annual exposure by including those entering the mines since 1972, in order to be able to estimate more precisely the risk for exposure groups in the ranges 10–30/30–60/60–90/90–120 WLM.

Data on French uranium miners' individual exposures to gamma irradiation and long lived ore dust are available since 1959 and we intend to study these two components, as well as radon exposure, on the cohort of miners entering the mines after 1959. This cohort will represent a population with low annual radon exposure (1–3 WLM per year); an exposure of 2 WLM is equivalent to an annual domestic exposure to radon gas of about 400 $Bq.m^{-3}$.

The analysis of these French uranium miners will be completed by an internal comparison analysis, as described in the BEIR IV report, taking into account factors such as age at first or last exposure, time elapsed since last exposure, and annual dose rate. This last point has been discussed by Darby *et al.* (1990) and Hornung *et al.* (1989), postulating an inverse dose-rate effect. Our study does not seem to favour this hypothesis.

### Conclusion

This first analysis of data from the French uranium miners has to be considered as one step in the study of cancer risk linked to low doses of radon. International collaboration with joint analysis of the data from the different cohorts or case-control studies will be more powerful than individual studies and will be the best approach to fixing limits of cancer risk linked to low radon exposures; most of the studies published in the past have focused on radon exposure at very high levels to which miners had been exposed in the years 1950–1960, when radon measurements were rare and the equilibrium factor often unknown. Consequently the individual exposures before 1960 have usually had to be reconstituted and may be less accurate than for the periods after 1960–1965, when systematic registration of radon measurements were implemented in most of the relevant countries. In France we have had complete systematic registration of the miners radon exposure since 1956, but at the end of 1985 the mean age of the young sub-cohort is still too young (mean age 55 years) for most of the potential risk of lung cancer to have been expressed. Follow-up over the next 5 to 10 years may give a more precise indication of the risk linked to very low exposures to radon and its decay products.

We gratefully acknowledge the assistance of Mr Bernhard and his team (COGEMA/CRPM) in the reconstruction of the dosimetric exposure of the miners, the advice of Messrs. Pradel and Zettwoog in charge of the radon measurements in the mines, and the efforts of the medical team of COGEMA in Razes who traced the deceased population and verified the causes of death. Helpful comments during our statistical analysis came from C. Hill and A. Auquier, Department of Statistical Medicine, Institut Gustave Roussy, Villejuif.

### References

ARCHER, V.E,. WAGONER, J.K. & LUNDIN, F.E. (1973). Lung cancer among uranium miners in the United States. *Health Phys.*, **25**, 351–371.

BEIR IV (1988). Health risks of radon and other internally deposited alpha-mitters. Committee on the biological effects of ionizing radiations, National Research Council, National Academy Press, Washington, D.C.

BRESLOW, N.E. & DAY, N.E. (1987). Statistical methods in cancer research, vol II, *The Design and Analysis of Cohort Studies*, IARC Scientific Publication no. 82.

CHAMEAUD, J., MASSE, R., MORIN, M. & LAFUMA, J. (1985). Lung cancer induction by radon daughters in rats, present state of the data on low dose exposures. In: *Proceedings of the International Conference on Occupational Radiation Safety in Mining*, **1**, 350–353. Canandian Nuclear Association, Toronto, Canada, H. Stocker editor.

COLEMAN, M.P., HERMON, C., & DOUGLAS, A. (1989). Person-years (PYRS). A Fortran Program for cohort study analysis, *IARC Internal report*. No. 89/006, Lyon.

CROSS, F.T., PALMER, R.F., FILIPY, R.E., DAGLE, G.E. & STUART, B.O. (1982). Carcinogenic effects of radon daughters, uranium ore dust and cigarette smoke in beagle dogs. *Health Phys*, **42**, 33–52.

DARBY, S.C. & DOLL, R. (1990). Radiation and exposure rate. *Nature*, **433**, 824.

GLIM. Statistical Software, Royal Statistical Society: London, UK.

HILL, C., REZVANI, A. & KRAMAR, A. (1984). Comparison of the mortality of a cohort to the mortality of a reference population. Number of expected deaths required to ensure a given power. *Rev. Epidém. et Santé Publ.*, **32**, 330–335.

HIRSCH, A. (1988). The fight against smoking in France. *Eur. Respir. J.*, **1**, 399–402.

HORNUNG, R.W. & MEINHARDT, T.J. (1987). Quantitative risk assessment of lung cancer in U.S. uranium miners. *Health Phys*,. **52**, 417–430.

HOWE, G.R., NAIR, R.C., NEWCOMBE, H.B., MILLER, A.B., BURCH, J.D. & ABBATT, J.D. (1987). Lung cancer mortality (1950–1980) in relation to radon daughter exposure in a cohort of workers at the Eldorado Port Radium Uranium Mine: possible modification of risk by exposure rate. *J. Natl Cancer Inst.*, **79**, 1255–1260.

IARC. (1988). Monographs on the evaluation of carcinogenic risks to humans. *Man Made Mineral Fibers and Radon*, **43**, IARC, Lyon.

ICRP 50. (1987). Lung cancer risk from indoor exposures to radon daughters. *International Commission on Radiobiological Protection*, **17**, 1, Pergamon Press.

KUNZ, E., SEVC, J., PLACEK, V. & HORACEK, J. (1979). Lung cancer in man in relation to different time distribution of radiation exposure. *Health Phys.*, **36**, 699–706.

LUBIN, J.H., QIAO, Y., TAYLOR, P.R., YAO, S.-X., SCHATZKIN, A,. MAO, B.-L., RAO, J.-Y., XUAN, X-Z. & LI, J.-Y. (1990). Quantitative evaluation of the radon and lung cancer association in a case control study of chinese tin mines. *Cancer Res.*, **50**, 174–180.

LUBIN, J.H. (1988). Models for the analysis of radon-exposed populations. *The Yale J. Biol Med.*, **61**, 195–214.

MULLER, J., WHEELER, W.C., GENTLEMAN, J.F., SURANYI, G. & KUSIAK, R. (1984). Study of mortality of Ontario Miners. In *Proceedings of the International Conference on Occupational Radiation Safety in Mining*, **1**, 335–343. Canadian Nuclear Association, Toronto, Canada, H. Stocker, editor.

MORRISON, H.I., SEMENCIW, R.M., MAO, Y. & WIGLE, D.T. (1988). Cancer mortality among a group of fluorspar miners exposed to radon progeny. *Am. J. Epidem.*, **128**, 1266–1275.

RADFORD, E.P. & ST. CLAIR RENARD, K.G.. (1984). Lung cancer in Swedish iron miners exposed to low doses of radon daughters. *N. Engl. J. Med.*, **310**, 1485–1494.

SAMET, J.M., PATHAK, D.R., MORGAN, M.V., KEY, C.R., VALDIVIA, A.A. & LUBIN, J.H. (1991). Lung cancer mortality and exposure to Rn progeny in a cohort of new-Mexico underground U miners. *Health Phys.*, **61**, 745–752.

SEVC, J., KUNZ, E., TOMASEK, L., PLACEK, V. & HORACEK, J. (1988). Cancer in man after exposure to Rn Daughters. *Health Phys.*, **54**, 27–46.

TIRMARCHE, M., BRENOT, J., PIECHOWSKI, J., CHAMEAUD, J. & PRADEL, J. (1984). The present state of an epidemiological study of uranium miners in France. In *Proceedings of the International Conference on Occupational Radiation Safety in Mining*, **1**, 344–349. Canadian Nuclear Association, Toronto, Canada, H. Stocker editor.

WAXWEILLER, R.J., ROSCOE, R.J., ARCHER, V.E., THUN, M.J., WAGONER, J.K. & LUNDIN, F.E. (1981). Mortality follow-up through 1977 of the white underground uranium miners cohort examined by the United States Public Health Service. In *Radiation Hazards in Mining: Control, Measurement and Medical Aspects*, 823–830. New York, Society of Mining Engineers of American Institute of Mining, Metallurgical and Petroleum Engineers, Gomez, M., editor.